# 超高效液相色谱-串联质谱测定畜肉中14种*β*-受体激动剂

**DOI:** 10.3724/SP.J.1123.2023.03008

**Published:** 2023-12-08

**Authors:** Jieqiong DONG, Jin XIAO, Xin ZHOU, Ning LI, Xuesong WANG, Junjie KANG

**Affiliations:** 1.唐山市农产品质量安全检验检测中心,唐山市食品药品综合检验检测中心,河北唐山063000; 1. Tangshan Agricultural Products Quality and Safety Inspection and Testing Center, Tangshan Food and Drug Comprehensive Inspection and Testing Center, Tangshan 063000, China; 2.遵化市畜牧水产技术服务中心,河北唐山063000; 2. Zunhua Livestock and Aquatic Products Technical Service Center, Tangshan 063000, China

**Keywords:** 超高效液相色谱-串联质谱, 一步式净化固相萃取柱, *β*-受体激动剂, 畜肉, ultra high performance liquid chromatography-tandem mass spectrometry (UHPLC-MS/MS), one-step purification solid-phase column, *β*-agonist, animal meat

## Abstract

在动物饲养过程中使用*β*-受体激动剂类药物可以显著提高猪、牛、羊等动物的瘦肉率。但是残留*β*-受体激动剂的食物被消费者食用后会危害身体健康。该文建立了超高效液相色谱-串联质谱快速检测畜肉中14种*β*-受体激动剂(克伦特罗、沙丁胺醇、莱克多巴胺、氯丙那林、特布他林、妥布特罗、溴布特罗、班布特罗、齐帕特罗、马布特罗、非诺特罗、福莫特罗、西马特罗、西布特罗)的方法,并对样品前处理和色谱条件进行了优化。样品加入乙酸铵缓冲液(pH 5.2)和*β*-盐酸葡萄糖醛苷酶/芳基硫酸酯酶,在(36±2) ℃水浴中酶解16 h后,放置至室温。经0.5%甲酸乙腈提取,加入NaCl粉末使乙腈和水相分层,取5 mL上层乙腈,使用一步式净化固相萃取柱进行净化,50 ℃氮气吹干后用0.4 mL 0.2%甲酸水溶液复溶,过0.22 μm微孔滤膜后上机分析。以乙腈和0.1%甲酸水溶液作为流动相进行梯度洗脱分析,经过Phenomenex Kinetex F5色谱柱分离后采用正离子扫描,多反应监测(MRM)模式进行检测,使用内标法和外标法定量。讨论了前处理过程中提取液的pH值、固相萃取柱种类、净化方式和复溶液的种类对提取效率的影响。采用超高效液相色谱-四极杆飞行时间质谱仪验证了一步式净化固相萃取柱的净化效果,证明了此类固相萃取柱能除去样品提取液中大部分的磷脂、鞘脂和甘油酯类物质。考察了仪器分析过程中色谱柱、流动相等影响因素。采用质谱和废液切换模式,既保证了样品中的杂质能及时从色谱柱中流出,又避免过多的杂质进入质谱。结果表明,14种*β*-受体激动剂在1.0~50 μg/L范围内线性关系良好,相关系数均大于0.99,方法的检出限为0.1~0.2 μg/kg,定量限为0.3~0.6 μg/kg。在低、中、高3个加标水平下,14种*β*-受体激动剂的平均回收率为70.25%~117.48%,相对标准偏差为0.63%~14.29%。该方法稳定性好,准确度高,适用于畜肉中多种*β*-受体激动剂残留的检测。

## Abstract (en)

The addition of *β*-agonists to animal feed can significantly improve the lean-meat rate of pigs, cattle, sheep, and other animals. However, the food residues of *β*-agonists are harmful to human health. When meat with *β*-agonist residues is consumed, poisoning symptoms such as palpitation, dizziness, and muscle tremors may develop, and damage to the cardiovascular system, liver, and kidney may occur. In this study, a method based on ultra high performance liquid chromatography-tandem mass spectrometry (UHPLC-MS/MS) was established for the rapid detection of 14 *β*-agonists (clenbuterol, salbutamol, ractopamine, clorprenaline, terbutaline, tulobuterol, bromobuterol, bambuterol, zilpaterol, mabuterol, fenoterol, arformoterol, cimaterol, and cimbuterol) in animal food sources. The sample pretreatment method and chromatographic conditions were optimized. The samples were hydrolyzed with *β*-glucuronidase hydrochloride/aryl sulfate esterase in ammonium acetate buffer (pH 5.2). Enzymatic hydrolysis was performed in a constant-temperature water bath ((36±2) ℃) oscillator for 16 h. The samples were cooled to room temperature and extracted with 0.5% formic acid acetonitrile. NaCl was added to separate the organic and aqueous phases, and 5 mL of the upper organic layer was purified using a one-step purification solid-phase extraction column. After drying with nitrogen at 50 ℃, the residue was dissolved in 0.4 mL of 0.2% formic acid aqueous solution. The samples were passed through a 0.22 μm filter and detected by UHPLC-MS/MS with gradient elution using acetonitrile and 0.1% formic acid aqueous solution as the mobile phases. The analytes were separated on a Phenomenex Kinetex F5 column and detected by positive-ion scanning in multiple-reaction monitoring (MRM) mode. Internal and external standard methods were used for quantitative analysis. The effects of the extract pH, solid-phase extraction column, purification method, and dissolved solution on the extraction efficiency were optimized during pretreatment. UHPLC-quadrupole time-of-flight MS was used to verify the purification effect of the one-step purification solid-phase extraction column, and the results indicated that this type of column could remove most of the phospholipids, sphingolipids, and glycerides in the sample extract. The factors influencing the different chromatographic columns and mobile phases were investigated. MS scanning was conducted in positive-ion mode with needle pump injection in mass-only mode, and the two daughter ions with the highest responses for each target were selected as the quantitative and qualitative ions. The declustering potential (DP) and collision energy (CE) of each ion were separately optimized in MRM mode. The switching mode of the mass spectrum and waste liquid was used, and the mobile phase was switched to waste liquid after all the target peaks were removed. These steps ensured that impurities in the sample flowed out of the column in a timely manner and that the effects of excessive impurities on the mass spectra were avoided. The 14 *β*-agonists showed good linear relationships in the range of 1.0-50 μg/L, with correlation coefficients of >0.99. The limits of detection (LODs) and quantification (LOQs) were in the range of 0.1-0.2 and 0.3-0.6 μg/kg, respectively. The average recoveries of the 14 *β*-agonists ranged from 70.25% to 117.48%, with relative standard deviations (RSDs) in the range of 0.63%-14.29% at low, medium, and high spiked levels. Pork, beef, and mutton samples were selected and analyzed using the developed method. The results were close to those of the national standard method, indicating that the method is accurate and reliable. Moreover, the proposed method has good stability and high accuracy; thus, it is suitable for the qualitative and quantitative determination of *β*-agonists in animal meat.

*β*-受体激动剂类药物,俗称“瘦肉精”,在临床医学中是一类用于治疗哮喘类疾病的药物,当把治疗剂量的5~10倍药量用在动物饲养环节中,就会产生营养再分配效应,能促进动物体内脂肪的分解代谢,加快蛋白质的合成速率,显著提高胴体的瘦肉率。当有“瘦肉精”残留的肉类食品被消费者食用后可能会引起心悸、头晕、肌肉震颤等中毒症状,对心血管和肝脏肾脏等造成一定的损伤^[1⇓-3]^。故我国农业农村部已禁止*β*-受体激动剂类药物在畜牧养殖行业的使用。食品安全监管机构和有关企业也将这类药物作为日常检测项目进行重点监督。

目前,检测*β*-受体激动剂类药物的方法主要包括:胶体金免疫层析法^[4]^、酶联免疫分析法^[5]^、气相色谱-质谱法^[6]^和液相色谱-质谱法^[7⇓⇓-10]^等。胶体金免疫层析法和酶联免疫分析法检测成本低,耗时短,适用于大批量样品的快速筛查,但是检测结果假阳性率高,不适用于确证检测。气相色谱-质谱法需要对样品先进行衍生化处理,过程繁琐。而液相色谱-质谱法准确性好、灵敏度高,不需要衍生化处理,还可以同时检测样品中多种类药物,在当前的兽药残留检测工作中应用最为广泛,我国现行有效的*β*-受体激动剂类药物的检测标准也以液相色谱-质谱法居多。

在前处理过程中,动物源性食品中的蛋白质和脂肪会随着目标化合物一起被提取出来,这些物质对目标化合物的检测会产生干扰,也会对仪器造成污染。在兽药残留检测中,通常采用固相萃取柱对提取液进行净化处理。整个使用过程包括活化、平衡、上样、淋洗、洗脱等,且各步骤所用溶液需要根据检测参数的不同进行相应优化。如王守英等^[11]^在测定动物尿液中23种*β*-受体激动剂时,选用了传统固相萃取柱对样品进行净化,整个优化过程要考虑多种情况,步骤较为繁琐。目前,在我国现行有效的*β*-受体激动剂的检测标准^[12,13]^中,无论是针对动物性食品、动物尿液还是饲料,多使用传统固相萃取柱,样品净化过程复杂且需要配制多种相关溶液,耗时较长,不适于大批量样品的同时检测。

本实验采用无需活化、直接上样的一步式净化固相萃取柱。相比于文献和国标法中使用的传统固相萃取柱,该柱在优化处理过程时需要考虑的处理条件较少,无需多次提取、浓缩,前处理过程更加简单快捷,且其对样品中的脂类物质和蛋白质等杂质的净化效果与传统固相萃取柱相当,也同样具有很好的重复性和灵敏度;同时还能够减少化学试剂的用量,安全环保,更加适用于批量样品的检测。

## 1 实验部分

### 1.1 仪器与试剂

AB Sciex Triple Quad 5500+超高效液相色谱-串联质谱联用仪、X500R超高效液相色谱-四极杆飞行时间质谱仪(美国AB SCIEX公司); KNORTH^®^ CAFS Clean-up LPAS食品中兽药多功能净化柱(北京科德诺思技术有限公司); TGL-20M台式高速冷冻离心机(湖南湘仪离心机仪器有限公司); ANPEL-DC24氮气吹干仪(上海安谱实验科技股份有限公司); 24位固相萃取装置(美国SUPELCO公司)。

标准物质:克伦特罗、沙丁胺醇、莱克多巴胺、氯丙那林、特布他林、妥布特罗、溴布特罗、班布特罗、齐帕特罗、马布特罗、非诺特罗、福莫特罗、西马特罗、西布特罗(100 μg/mL,天津阿尔塔科技有限公司)。

内标物质:克伦特罗-D_9_、莱克多巴胺-D_3_、沙丁胺醇-D_3_、氯丙那林-D_7_、西马特罗-D_7_、特布他林-D_9_(100 μg/mL,天津阿尔塔科技有限公司)。

乙酸铵、氢氧化钠、氯化钠(分析纯,国药集团化学试剂有限公司); *β*-盐酸葡萄糖醛苷酶/芳基硫酸酯酶(美国Sigma公司);甲醇、乙腈、甲酸(色谱纯,德国Merck公司)。

### 1.2 标准溶液的配制

用甲醇将各标准物质和内标物质分别稀释成质量浓度为10 μg/mL的标准储备液和内标储备液,避光保存于-20 ℃冰箱。

分别取适量上述标准储备液和内标储备液混匀,用10%甲醇水稀释成100 ng/mL的混合标准溶液和混合内标溶液,现用现配。

### 1.3 样品前处理

称取2.0 g(精确至0.01 g)均质试样,置于50 mL塑料离心管中,加入0.2 mol/L乙酸铵溶液(pH 5.2)8.0 mL,再加入*β*-盐酸葡萄糖醛苷酶/芳基硫酸酯酶50 μL和100 ng/mL的混合内标溶液40 μL,涡旋混匀后,于(36±2) ℃水浴16 h。

样品酶解后放置至室温,加入10 mL 0.5%甲酸乙腈,振荡提取10 min,加入3.0~3.5 g NaCl粉末,涡旋混匀,8000 r/min离心5 min,吸取5 mL上层清液过固相萃取柱,洗脱液在50 ℃条件下氮吹至近干,残留物用0.4 mL 0.2%甲酸水溶液复溶,超声10 min,涡旋混匀后过0.22 μm的微孔滤膜,供UHPLC-MS/MS分析。

### 1.4 分析条件

#### 1.4.1 色谱条件

色谱柱:Phenomenex Kinetex F5色谱柱(100 mm×2.1 mm, 2.6 μm);柱温40 ℃;流动相A: 0.1%甲酸水溶液,流动相B:乙腈;梯度洗脱程序:0~1.0 min, 5%B~10%B; 1.0~7.0 min, 10%B~70%B; 7.0~8.0 min, 70%B~100%B; 8.0~9.0 min, 100%B; 9.0~9.1 min, 100%B~5%B; 9.1~10.0 min, 5%B。流速:0.25 mL/min,进样量:2 μL。

#### 1.4.2 质谱条件

电喷雾离子源正离子扫描方式(ESI^+^),MRM模式;高纯氮气,气帘气压力(CUR): 241.3 kPa(35 psi);喷雾气压力(GS1): 344.7 kPa(50 psi);辅助加热气压力(GS2): 344.7 kPa(50 psi);离子源温度:550 ℃;离子化电压:+5500 V。14种*β*-受体激动剂的保留时间、定量与定性离子对、去簇电压(DP)和碰撞能量(CE)见表1。

**表1:** 14种*β*-受体激动剂及内标的保留时间和质谱参数

Compound	Retention time/min	Parent ion (*m/z*)	Daughter ions (*m/z*)	DPs/V	CEs/eV	IS
Clenbuterol (克伦特罗)	4.23	277.1	203.0^*^/132.1	46/51	23/37	clenbuterol-D_9_
Salbutamol (沙丁胺醇)	2.22	240.1	148.1^*^/166.0	41/31	25/19	salbutamol-D_3_
Ractopamine (莱克多巴胺)	3.85	302.1	164.0^*^/284.0	61/66	21/27	ractopamine-D_3_
Clorprenaline (氯丙那林)	3.69	214.1	119.1^*^/154.0	41/41	37/23	clorprenaline-D_7_
Terbutaline (特布他林)	2.24	226.1	152.2^*^/107.0	31/31	21/39	terbutaline-D_9_
Tulobuterol (妥布特罗)	4.09	228.1	154.1^*^/119.0	51/36	23/39	clorprenaline-D_7_
Bromobuterol (溴布特罗)	4.46	367.1	293.0^*^/349.0	66/56	25/17	ractopamine-D_3_
Bambuterol (班布特罗)	4.50	368.1	294.1^*^/312.2	66/56	27/19	-
Zilpaterol (齐帕特罗)	2.19	262.1	244.1^*^/185.1	51/36	17/33	ractopamine-D_3_
Mabuterol (马布特罗)	4.76	311.1	237.1^*^/217.0	51/61	23/35	clorprenaline-D_7_
Fenoterol (非诺特罗)	3.11	304.2	107.0^*^/135.1	40/40	35/23	cimaterol-D_7_
Arformoterol (福莫特罗)	4.18	345.2	149.2^*^/121.0	40/40	25/37	clorprenaline-D_7_
Cimaterol (西马特罗)	2.41	220.1	202.2^*^/143.1	26/31	13/31	cimaterol-D_7_
Cimbuterol (西布特罗)	3.03	234.1	160.2^*^/143.1	26/36	19/35	salbutamol-D_3_
Clenbuterol-D_9_ (克伦特罗-D_9_)	4.23	286.3	204.1	65	25	
Salbutamol-D_3_ (沙丁胺醇-D_3_)	2.22	243.2	151.2	41	24	
Ractopamine-D_3_ (莱克多巴胺-D_3_)	3.85	305.2	167.2	61	23	
Cimaterol-D_7_ (西马特罗-D_7_)	2.41	227.0	161.0	40	23	
Terbutaline-D_9_ (特布他林-D9)	2.24	235.2	153.1	40	20	
Clorprenaline-D_7_ (氯丙那林-D_7_)	3.69	221.0	155.1	41	23	

DP: declustering potential; CE: collision energy; * quantitative ion.

## 2 结果与讨论

### 2.1 色谱和质谱条件优化

#### 2.1.1 色谱条件的优化

实验对比了Waters ACQUITY UPLC BEH C_18_(100 mm×2.1 mm, 1.7 μm)、Waters ACQUITY UPLC HSS T3(100 mm×2.1 mm, 1.8 μm)和Phenomenex Kinetex F5(100 mm×2.1 mm, 2.6 μm)色谱柱对这14种*β*-受体激动剂的分离性能。发现BEH C_18_色谱柱对沙丁胺醇的保留能力较弱,保留时间较短且峰形展宽拖尾,通过改变流动相的种类和梯度也无法改善。对比HSS T3色谱柱和F5色谱柱,14种*β*-受体激动剂在两种色谱柱上均能较好分离,保留时间适宜且峰形尖锐对称。通过各目标物响应对比后发现,非诺特罗和齐帕特罗在F5色谱柱上的响应值比HSS T3色谱柱上高约30%,其他目标物在两种色谱柱上响应相近。故最终选择Phenomenex Kinetex F5色谱柱。

以乙腈为有机相,对比了0.1%甲酸水溶液和含5 mmol/L甲酸铵的0.1%甲酸水溶液作为水相的区别。结果表明:在两种流动相条件下,各化合物均峰形良好,各目标物峰面积也相近。但考虑到盐类物质如果操作不当会造成色谱柱和管路的堵塞,最后选择0.1%甲酸水溶液作为水相。

#### 2.1.2 质谱条件的优化

在仪器Mass Only模式下,用针泵注射进样的方式,在正离子模式下扫描,每个目标物选择响应最高的两个子离子分别作为定量离子和定性离子。然后在MRM模式下分别优化每个离子的去簇电压和碰撞能量,优化后的参数见表1。最后,采用质谱和废液切换模式,在所有目标物从色谱柱上洗脱后,将流动相切换到废液,这样既保证了样品中的杂质能及时从色谱柱中流出,又避免过多的杂质进入质谱,可以起到保护仪器的作用。

### 2.2 前处理条件的优化

#### 2.2.1 提取液的优化

以阴性样品为实验对象,制作空白加标样品。在样品中加入乙腈提取液后,用甲酸和NaOH溶液调节提取液的pH值(4.0~11.0),考察不同pH值对14种*β*-受体激动剂提取效果的影响(见图1)。结果表明,提取液的pH值对*β*-受体激动剂的提取有一定的影响,当pH为4.0~6.0时,各*β*-受体激动剂的响应值达到最高且结果相近。但是用pH计来调节每一个样品的pH值耗时较长,不适用于批量样品的处理。故选择直接加入甲酸酸化乙腈的方式来调整提取液的pH值。因此实验进一步考察了0.5%、1.5%、3.0%和5.0%的甲酸乙腈作为提取液时14种*β*-受体激动剂提取的提取效果,结果见图2。结果表明,4种甲酸乙腈对14种*β*-受体激动剂提取效果相近,当提取液为0.5%甲酸乙腈时,莱克多巴胺、特布他林、西布特罗、非诺特罗、马布特罗、班布特罗、溴布特罗和福莫特罗的提取效率稍高,当提取液为1.5%的甲酸乙腈时,克伦特罗、沙丁胺醇、氯丙那林、西马特罗、齐帕特罗和妥布特罗的提取效率稍高。综合各目标物的提取效率和实验成本,实验最终确定使用0.5%甲酸乙腈作为提取液。

**图1:**
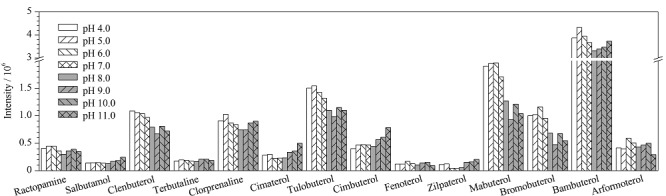
提取液的pH值对14种*β*-受体激动剂提取效率的影响

**图2:**
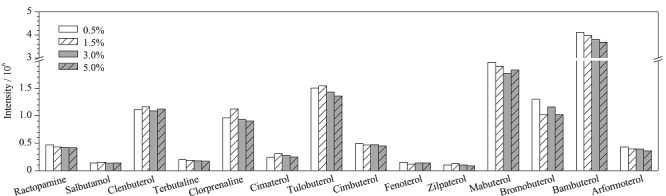
不同体积分数的甲酸乙腈溶液对14种*β*-受体激动剂提取效率的影响

#### 2.2.2 固相萃取柱的选择

实验分别对比了4种一步式净化固相萃取柱Captiva EMR-Lipid(SPE-A)、KNORTH^®^ CAFS Clean-up LPAS食品中兽药多功能净化柱(SPE-B)、OMNI Creole Prime HLB(SPE-C)和Oasis^®^ PRiME HLB(SPE-D)与不使用固相萃取柱的情况下空白添加样品中各*β*-受体激动剂的响应,结果见图3。由图3可知,使用固相萃取柱的样品中大部分*β*-受体激动剂的响应比未使用的样品响应更高。说明这14种*β*-受体激动剂均能顺利通过固相萃取柱,并未被柱中填料吸附。并且经过固相萃取柱除杂后,减轻了仪器分析过程中杂质对目标物的干扰,使响应增强。

**图3:**
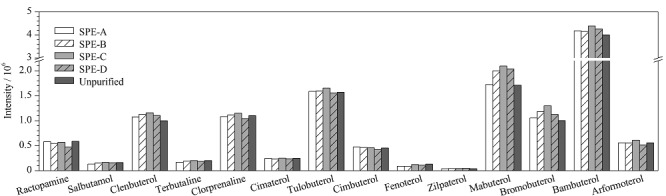
不同固相萃取柱对14种*β*-受体激动剂提取效率的影响

为检验一步式净化固相萃取柱的净化效果,采用超高效液相色谱-四极杆飞行时间质谱仪对分别使用4种一步式固相萃取柱的样品溶液进行磷脂、鞘脂和甘油酯含量检测,并与不使用固相萃取柱的样品进行对比,总离子流色谱图如图4所示。可以看出,一步式净化固相萃取柱能有效除去样品提取液中大部分的磷脂、鞘脂和甘油酯类干扰物质。以使用固相萃取柱的样品中磷脂、鞘脂和甘油酯的含量与未净化样品的比值进行比较,发现在本实验的前处理方法下,SPE-A、SPE-B、SPE-D柱净化效果更好,均能除去样品提取液中98%以上的磷脂、鞘脂和甘油酯类物质。

**图4:**
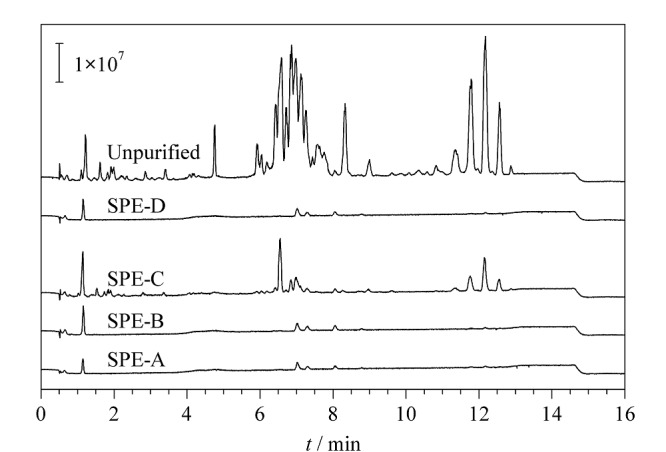
不同净化方式下样品中磷脂、鞘脂和甘油酯类 物质的总离子流色谱图

最后,综合各目标化合物的响应强度、净化效果以及固相萃取柱价格等方面的考虑,选择净化效果好且价格较低的SPE-B柱对提取液进行净化。

#### 2.2.3 净化方式的选择

根据文献^[14⇓-16]^报道,一步式净化固相萃取柱的上样溶液通常选择70%~85%乙腈水溶液,因此,本实验对比了以下3种处理方式对目标物仪器分析的影响:(1)取5 mL乙腈层上清液与1 mL超纯水混合后过柱,氮吹至近干。(2)取5 mL乙腈层上清液与1 mL超纯水混合后过柱,氮吹至1 mL(不足1 mL时用蒸馏水定容到1 mL); (3)取5 mL乙腈层上清液直接上样后,氮吹至近干。结果表明:(1)中各参数的响应值均低于(2)和(3),可能是因为氮吹时间过长,造成了目标物的损失。(3)中沙丁胺醇定性离子中的杂峰峰面积比(2)小,其他参数(2)与(3)结果类似,且(3)的氮吹时长比(2)短,操作也更简便,故最终选择处理方式(3)。

#### 2.2.4 定容溶液的选择

分别用0.2%甲酸水溶液、10%乙腈水溶液、20%乙腈水溶液、30%乙腈水溶液稀释标准品溶液,考察溶剂对目标物仪器分析的影响。结果表明,用0.2%甲酸水溶液或10%乙腈水溶液稀释的标准溶液,各目标物响应高,色谱峰形尖锐、对称,重复性好。但随着乙腈体积分数的增大,所有药物峰展宽越来越严重,响应也变得不稳定,峰面积重复性变差。故选择0.2%甲酸水溶液、10%乙腈水溶液为样品氮气吹干后的复溶液做进一步验证。

将空白加标样品进行前处理,氮气吹干后分别用0.2%甲酸水溶液和10%乙腈水溶液定容,超声10 min,涡旋过膜后上机分析。结果表明:两种定容溶液对仪器分析结果影响不大,各目标物响应相近。但是在定容后过0.22 μm的微孔滤膜时,用0.2%甲酸水溶液定容的样品更加澄清且过膜阻力更小,推测可能是10%乙腈水溶液能将氮气吹干后附着在管壁的残留脂类物质溶解下来,使定容溶液呈乳浊液,以致过膜时阻力更大。因此,本实验最终选择0.2%甲酸水溶液作为定容溶液。

### 2.3 方法学评价

#### 2.3.1 基质效应

基质效应(ME)是影响检测结果准确性的一个重要因素,也是评价前处理方法好坏的一个重要指标。基质效应越强,方法的准确性越低^[17,18]^。本实验以猪肉、牛肉和羊肉阴性样品为基质,用外标法和内标法分别绘制基质匹配标准曲线,根据文献^[19]^报道的方法,通过公式ME=(基质标准曲线斜率/溶剂标准曲线斜率-1)×100%来计算14种*β*-受体激动剂的基质效应,用以评价不同基质中的干扰物对目标物的影响以及内标法降低基质效应的效果。当基质效应小于20%时,为弱基质效应;20%~50%时为中等基质效应;大于50%时为强基质效应。结果表明,在3种基质中,当使用外标法时,大部分目标物会产生中等基质效应甚至强基质效应,但使用内标法后,大部分目标物为弱基质效应,表明在这14种*β*-受体激动剂的检测中,使用内标法可以有效减小基质效应。

#### 2.3.2 标准曲线、检出限和定量限

配制14种*β*-受体激动剂的系列标准溶液,班布特罗以目标化合物的定量离子峰面积为纵坐标(*Y*),相应的质量浓度为横坐标(*X*, ng/mL),绘制标准曲线。其余目标物以目标化合物的定量离子峰面积与内标峰面积之比为纵坐标(*y*),相应的质量浓度之比为横坐标(*x*),绘制标准曲线。以阴性样品为基质,做标准添加样品,以3倍信噪比(*S/N*)对应的加标浓度作为检出限,以10倍信噪比对应的加标浓度作为定量限。结果如表2所示,14种*β*-受体激动剂在1.0~50 μg/L范围内线性关系良好,相关系数(*r*)>0.99,方法的检出限为0.1~0.2 μg/kg,定量限为0.3~0.6 μg/kg。现行有效的动物源性食品中*β*-受体激动剂检测的国标方法^[13]^中,检出限为0.25 μg/kg,定量限为0.5 μg/kg,说明该方法与国标方法灵敏度相当,能够满足样品中*β*-受体激动剂的定量检测需求。

**表2:** 14种*β*-受体激动剂的线性范围、线性方程、相关系数、检出限和定量限

Compound	Linear range/(μg/L)	Linear equation	*r*	LOD/(μg/kg)	LOQ/(μg/kg)
Clenbuterol	1.0-50	*y*=4.74×10^-2^*x*-3.00×10^-4^	0.9996	0.1	0.3
Salbutamol	1.0-50	*y*=1.18*x*-1.06×10^-2^	0.9997	0.1	0.3
Ractopamine	1.0-50	*y*=1.09*x*-1.34×10^-2^	0.9999	0.1	0.3
Clorprenaline	1.0-50	*y*=1.40*x*+9.75×10^-3^	0.9994	0.1	0.3
Terbutaline	1.0-50	*y*=1.74*x*+5.79×10^-2^	0.9985	0.1	0.3
Tulobuterol	1.0-50	*y*=1.76*x*-2.19×10^-2^	0.9993	0.1	0.3
Bromobuterol	1.0-50	*y*=1.91*x*-6.44×10^-2^	0.9994	0.1	0.3
Bambuterol	1.0-50	*Y*=6.65×10^5^*X*-1.47×10^4^	0.9994	0.1	0.3
Zilpaterol	1.0-50	*y*=4.22×10^-1^*x*-3.16×10^-2^	0.9982	0.1	0.3
Mabuterol	1.0-50	*y*=1.97*x*-4.13×10^-3^	0.9996	0.1	0.3
Fenoterol	1.0-50	*y*=1.14*x*+8.34×10^-2^	0.9994	0.2	0.6
Arformoterol	1.0-50	*y*=8.14×10^-1^*x*-2.46×10^-3^	0.9996	0.1	0.3
Cimaterol	1.0-50	*y*=2.02*x*+3.29×10^-2^	0.9995	0.1	0.3
Cimbuterol	1.0-50	*y*=1.23*x*-3.39×10^-2^	0.9984	0.2	0.6

*y*: ratio of peak area of compound to internal standard; *x*: ratio of mass concentration of compound to internal standard; *Y*: peak area; *X*: mass concentration, ng/mL.

#### 2.3.3 加标回收率与精密度

选择阴性猪肉、牛肉、羊肉样品,分别添加低、中、高3个不同水平的混合标准溶液。按1.3.1节进行前处理,每个水平重复测定6次,其中班布特罗用外标法定量,其余目标物用内标法定量,计算回收率和相对标准偏差(RSD)。结果如表3所示,14种*β*-受体激动剂的平均回收率为70.25%~117.48%,相对标准偏差为0.63%~14.29%。

**表3:** 14种*β*-受体激动剂在实际样品中的加标回收率和相对标准偏差(*n*=6)

Compound	Spiked level/ (μg/kg)	Recoveries/%				RSDs/%		
		Pork	Beef	Mutton		Pork	Beef	Mutton
Clenbuterol	0.3	87.39	98.99	100.39		6.38	4.94	9.04
	1	103.11	99.39	104.37		12.64	7.92	7.91
	5	97.03	99.75	103.53		10.43	7.23	6.08
Salbutamol	0.3	92.26	101.39	96.90		11.08	1.21	6.87
	1	106.43	108.12	98.85		3.18	5.53	4.74
	5	100.83	106.67	99.91		5.90	4.13	2.19
Ractopamine	0.3	96.03	99.13	98.72		7.42	1.46	6.98
	1	101.07	100.17	96.52		2.93	8.09	5.99
	5	86.82	97.99	97.62		12.07	5.95	5.39
Clorprenaline	0.3	95.49	100.54	99.09		6.73	1.01	6.96
	1	106.00	107.30	100.61		3.28	2.51	2.05
	5	102.57	104.82	101.23		2.68	4.28	3.75
Terbutaline	0.3	85.52	91.34	95.30		4.56	7.21	7.43
	1	94.42	88.04	89.66		12.64	7.92	7.91
	5	93.09	86.94	79.63		10.43	7.23	6.08
Tulobuterol	0.3	89.27	103.05	71.79		9.74	0.76	5.36
	1	117.48	112.60	114.55		4.32	4.82	2.01
	5	84.61	110.70	113.24		2.49	3.96	2.22
Bromobuterol	0.3	100.78	96.42	88.00		6.09	0.63	8.27
	1	94.33	93.83	103.65		13.54	10.23	3.50
	5	73.80	80.48	80.59		11.68	13.03	4.28
Bambuterol	0.3	72.24	88.17	77.30		7.02	9.51	11.49
	1	80.51	84.02	98.89		3.15	8.06	2.01
	5	71.78	78.01	76.40		2.63	2.16	3.55
Zilpaterol	0.3	78.60	89.75	74.27		13.96	3.65	5.37
	1	95.45	95.29	96.43		7.54	10.02	7.68
	5	94.67	95.27	100.01		12.74	7.40	8.98
Mabuterol	0.3	91.54	75.53	89.02		4.14	2.47	4.78
	1	88.31	100.74	104.17		6.18	7.89	10.80
	5	70.25	88.84	83.17		3.16	6.89	6.28
Fenoterol	0.6	74.95	71.59	91.08		8.16	5.71	8.93
	1	94.23	96.52	112.20		12.24	10.58	14.06
	5	113.83	86.31	91.83		12.00	8.24	14.29
Arformoterol	0.3	87.22	96.80	101.48		4.92	1.26	12.13
	1	115.29	113.65	114.55		7.90	10.68	3.89
	5	96.37	117.41	74.19		11.69	13.99	3.96
Cimaterol	0.3	93.57	96.68	76.54		6.52	9.56	3.72
	1	102.92	115.92	106.78		8.63	8.29	2.01
	5	111.46	95.52	99.27		13.92	7.53	7.12
Cimbuterol	0.6	72.85	82.41	86.17		5.74	12.78	6.64
	1	97.78	102.69	104.06		10.81	10.86	8.34
	5	102.06	84.34	95.92		14.00	12.31	5.54

### 2.4 实际样品的测定

为了进一步验证方法的可行性和准确性,选取经国标方法^[13]^测定含有克伦特罗的猪肉样品(3.6 μg/kg)、牛肉样品(5.7 μg/kg),以及含有沙丁胺醇的羊肉样品(2.8 μg/kg),用所建方法对这3份样品进行检测,定性结果与标准方法相同。定量结果显示,猪肉样品中克伦特罗的含量为3.9 μg/kg,牛肉样品中克伦特罗的含量为5.5 μg/kg,羊肉样品中沙丁胺醇的含量为3.0 μg/kg,结果均与国标方法接近,说明本方法准确可靠,可以用于畜肉中*β*-受体激动剂的定性和定量测定。

## 3 结论

本文建立了固相萃取-超高效液相色谱-串联质谱同时测定畜肉中14种*β*-受体激动剂的方法。采用一步式净化固相萃取柱,既能有效去除样品中的干扰物质,又简化了固相萃取的操作,节省了实验时间。同时利用同位素内标法有效降低了实际样品的基质效应。此方法简单快捷,重复性好,准确度高,溶剂消耗量少,适合批量样品中*β*-受体激动剂残留的检测。
